# Impacts of zero-fare transit policy on health and social determinants: protocol for a natural experiment study

**DOI:** 10.3389/fpubh.2024.1458137

**Published:** 2024-10-21

**Authors:** Amanda Grimes, Jannette Berkley-Patton, Jenifer E. Allsworth, Joseph S. Lightner, Keith Feldman, Brent Never, Betty M. Drees, Brian E. Saelens, Tiffany M. Powell-Wiley, Lauren Fitzpatrick, Carole Bowe Thompson, Madison Pilla, Kacee Ross, Chelsea Steel, Emily Cramer, Eric Rogers, Cindy Baker, Jordan A. Carlson

**Affiliations:** ^1^School of Nursing and Health Studies, University of Missouri-Kansas City, Kansas City, MO, United States; ^2^Department of Biomedical and Health Informatics, School of Medicine, University of Missouri-Kansas City, Kansas City, MO, United States; ^3^Department of Pediatrics, School of Medicine, University of Missouri-Kansas City, Kansas City, MO, United States; ^4^Health Services and Outcomes Research, Children's Mercy Kansas City, Kansas City, MO, United States; ^5^Bloch School of Management, University of Missouri-Kansas City, Kansas City, MO, United States; ^6^Department of Internal Medicine, University of Missouri-Kansas City School of Medicine, Kansas City, MO, United States; ^7^Graduate School of the Stowers Institute for Medical Research, Kansas City, MO, United States; ^8^Department of Internal Medicine, University Health, Kansas City, MO, United States; ^9^Seattle Children's Research Institute, Seattle, WA, United States; ^10^Department of Pediatrics, University of Washington School of Medicine, Seattle, WA, United States; ^11^Social Determinants of Obesity and Cardiovascular Risk Laboratory, Cardiovascular Branch, Division of Intramural Research, National Heart, Lung, and Blood Institute, and Intramural Research Program, National Institute on Minority Health and Health Disparities, National Institutes of Health, Bethesda, MD, United States; ^12^Center for Children’s Healthy Lifestyles and Nutrition, Kansas City, MO, United States; ^13^Children’s Mercy, Kansas City, MO, United States; ^14^The University of Kansas Medical Center, Kansas City, KS, United States; ^15^BikeWalkKC, Kansas City, MO, United States; ^16^Kansas City Area Transportation Authority, Kansas City, MO, United States

**Keywords:** zero-fare transit, natural experiment, physical activity, social determinants of health, transportation

## Abstract

Population-level efforts are needed to increase levels of physical activity and healthy eating to reduce and manage chronic diseases such as obesity, cardiovascular disease, and type 2 diabetes. Interventions to increase public transit use may be one promising strategy, particularly for low-income communities or populations of color who are disproportionately burdened by health disparities and transportation barriers. This study employs a natural experiment design to evaluate the impacts of a citywide zero-fare transit policy in Kansas City, Missouri, on ridership and health indicators. In Aim 1, comparison to 9 similar cities without zero-fare transit is used to examine differential changes in ridership from 3 years before to 4 years after the adoption of zero-fare. In Aim 2, Kansas City residents are being recruited from a large safety net health system to compare health indicators between zero-fare riders and non- riders. Longitudinal data on BMI, cardiometabolic markers, and economic barriers to health are collected from the electronic health record from 2017 to 2024. Cross-sectional data on healthy eating and device-measured physical activity are collected from a subsample of participants as part of the study procedures (*N* = 360). Numerous baseline characteristics are collected to account for differences between Kansas City and comparison city bus routes (Aim 1) and between zero-fare riders and non-riders within Kansas City (Aim 2). Evidence on how zero-fare transit shapes population health through mechanisms related to improved economic factors, transportation, physical activity, and healthy eating among low-income groups is expected.

## 1 Introduction

Wide-reaching efforts are needed to increase population levels of physical activity and healthy eating in low-income groups for obesity and type 2 diabetes prevention/control. Some evidence suggests public transit can benefit health by creating opportunities for physical activity (e.g., walking to/from the bus) and improving access to healthy food and health services (1–7). Thus, approaches aiming to increase the reach and use of public transit have promise for improving population health. Transit-based opportunities may also support health equity given groups that have been economically or historically marginalized experience significant health disparities and rely more on public transit than their counterparts (8–12).

Previous research on the health impacts of transit policy/environmental changes has primarily focused on increasing access to transit via new or improved transit lines, such as the expansion of a bus line to rapid transit or construction of a new light rail line (13–17). Findings have been mixed. Some findings indicated no overall effect on transit use or overall physical activity (15–18), whereas others showed an increase in the proportion of nearby residents using transit (15), more walking around transit stops (19), improvements in overall physical activity and body mass index (BMI) among transit users (15), and a decrease in health care costs among some individuals (18). Gaps in previous research are that the type of policy or environmental changes investigated have been limited in scope (e.g., limited geographical coverage, only addressing new/expanded transit lines), and few studies have investigated impacts on economic barriers to health (18, 20) or health markers other than physical activity (15). Additionally, few studies have evaluated whether increases in transit use may correspond with unintended negative outcomes such as increased crime or pedestrian-related crashes (21–24).

Fare-free transit has been primarily studied outside the U.S., particularly for city-wide initiatives. Several studies have been conducted examining the city-wide, fare-free transit in Tallinn, Estonia (25–28). An increase in transit mode (25, 26) share and demand (27), a decrease in car use (25, 26), and increased mobility for lower-income groups (28) were associated with the fare-free policy. However, these studies are limited by the study design as there were no prospective studies and no studies included a control group. Studies on fare-free policies for specific populations (i.e., older adults, women) have found increases in physical activity among older adults (29–31), and increased access to jobs, improved income, and access to quality healthcare for women (20). To our knowledge, no studies have investigated the impacts of fare-free transit on BMI, obesity, or other cardiometabolic health markers.

**Figure 1:**
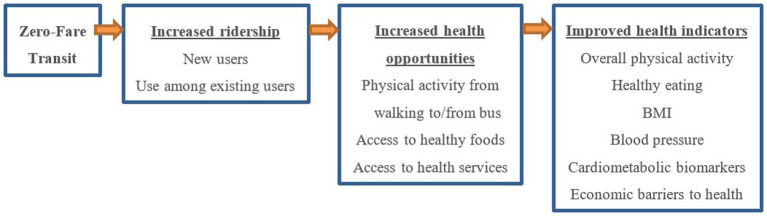
Conceptual model of hypothesized impacts of zero-fare bus transmit.

The current study aims to investigate one US city’s novel transit policy that eliminates bus fares for all users, referred to as zero-fare transit. In 2020, Kansas City, MO (KCMO), became the first major city in the U.S. to adopt an ongoing citywide zero-fare policy. This transit intervention differed from prior interventions in that it comprised a system-wide change largely intended to benefit low-income groups. As shown in Figure 1, zero-fare is posited to support increased ridership by providing a financial incentive (cost savings) for non-transit users to switch from using private vehicles to using the city bus for a portion of their trips (i.e., mode shift) and for existing transit users to use the city bus more often (i.e., increased mobility/access). Zero-fare riders may experience cardiometabolic health benefits through increased physical activity from walking to/from bus stops, increased access to health-related opportunities (e.g., places for engaging in healthy eating and active living, places for receiving health services), and reduced economic barriers to health (e.g., having sufficient money for transportation, medications, and nutrition). This publication presents the sample, design, and procedures for the Zero-Fare Bus Transit (ZBT) evaluation study. The ZBT study is testing the following aims:

1. To compare changes in bus ridership between Kansas City (zero-fare city) and non-zero-fare city bus routes.
2. To compare health indicators between zero-fare riders and non-riders.

## 2 Methods

### 2.1 Overview, design, and setting

**Figure 2:**
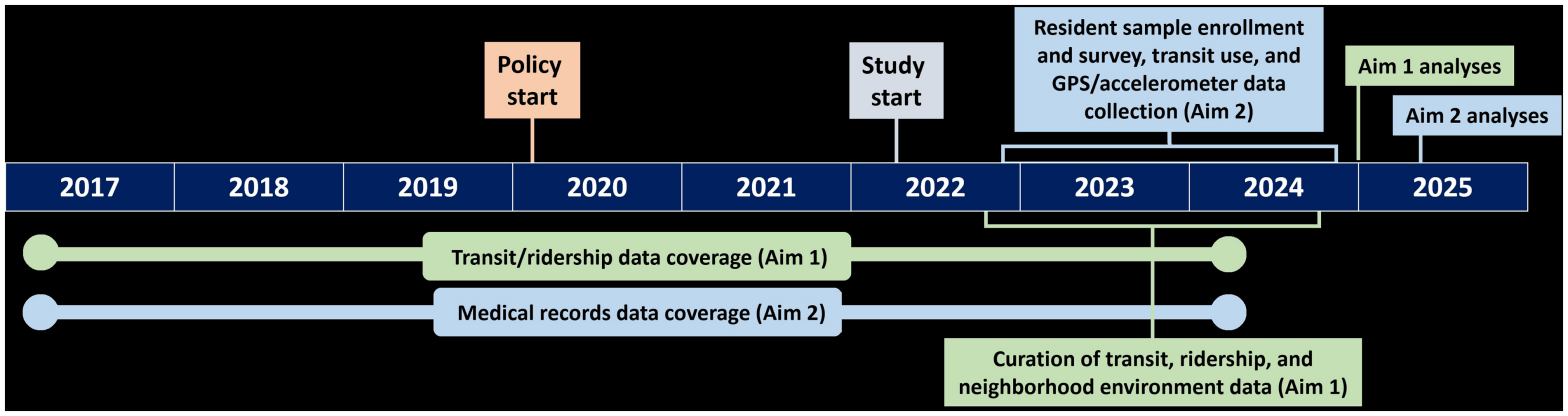
Timeline of the zero-fare transit study.

The ZBT study uses a quasi-experimental design. In Aim 1, longitudinal data from transit agencies are being used to compare ridership between KCMO bus routes (cases) and bus routes from comparison cities (controls). In Aim 2, KCMO residents are being enrolled to compare health indicators between zero-fare riders (i.e., those who use zero-fare bus transit; cases) and non-riders (controls) using longitudinal (i.e., BMI [primary outcome], biomarkers, economic barriers) and cross-sectional (i.e., physical activity, healthy eating) data. Given the quasi-experimental design, numerous neighborhood environment and transit characteristics (Aims 1–2) and participant characteristics (Aim 2) are being collected to account for baseline differences between the cases and controls. The study timeline (Figure 2) includes data spanning from 3 years prior to zero-fare to 4 years after. The 3-year baseline period helps show temporal trajectories in study variables prior to zero-fare and the 4-year follow up helps assess sustained impacts. The study was approved by the Institutional Review Boards at the sponsoring institutions and all study participants provided informed consent.

The zero-fare policy was adopted in March 2020 by the city of KCMO, which has a population of 491,158 residents and is the 6th largest city in the Midwest and 5th most economically and racially segregated city in the U.S. (32). As a result, communities in KCMO with a large proportion of non-White or Hispanic residents have a life expectancy up to 17 years lower than other communities (33). In 2019, the Kansas City Area Transportation Authority (KCATA) serviced 53 bus lines and an average daily weekday ridership of 33,000 trips. A majority of its service is provided within KCMO, with some routes extending to other cities in the metropolitan area. According to a 2019 KCATA Customer Survey, 44.5% of riders identified as Black, 28.5% as White non-Hispanic, and 9.3% as Hispanic; 67% did not have a working vehicle; 45% were between 13 and 32 years of age and 23% were between 33 and 42 years of age; 64% were male; and 77.1% had an annual income <$30 K. The city’s motivation to move to zero-fare was largely to improve transportation access, equity, and economic benefits among low-income communities, while also improving transit services by allowing for faster boarding and operations through fare elimination. All buses became zero-fare except for on-demand buses and a small number of micro-mobility services located outside of KCMO.

### 2.2 Sample and recruitment

#### 2.2.1 Ridership sample (Aim 1)

In partnership with the KCATA, the study team identified 9 cities that did not have a zero-fare policy and were comparable to KCMO across a number of demographic, transit, and climate characteristics (Table 1).

**Table 1:** Characteristics of cities included in the ZBT study.

	Population characteristics^a^							Transit characteristics^b^				Weather characteristics^c^		
City	City population	Metropolitan population	% White non-Hispanic	% Black non-Hispanic	% in poverty	% ride public transit to work	Mean population per sq. mile	Service population	Vehicles operated	Total routes operated	Mean daily ridership trips	Days >1 inch precipitation	Days <32° F^d^	Days >90° F^d^
Kansas City, MO^e^	486 K	2.12 M	55.2%	27.9%	16.1%	2.6%	1615	789 K	171	53	33.0 K	15	30	51
Austin, TX	951 K	2.11 M	48.3%	7.4%	13.2%	3.5%	3006	1,30 M	315	103	84.7 K	11	0	143
Cincinnati, OH	301 K	2.20 M	48.2%	42.0%	26.3%	7.1%	3974	745 K	298	47	36.2 K	18	18	38
Columbus, OH	879 K	2.08 M	55.1%	28.6%	19.5%	3.1%	4116	1,06 M	269	45	51.6 K	8	28	31
El Paso, TX	680 K	840 K	12.8%	3.2%	19.1%	1.6%	2627	747 K	125	62	21.2 K	0	0	128
Indianapolis, IN	864 K	2.03 M	54.5%	28.2%	18.0%	1.8%	2455	928 K	156	31	25.3 K	13	31	26
Louisville, KY	618 K	1.26 M	64.5%	23.7%	15.2%	2.9%	1469	807 K	182	39	27.3 K	21	10	80
Milwaukee, WI	595 K	1.58 M	35.1%	38.3%	25.4%	7.3%	6001	948 K	307	30	67.7 K	10	54	10
Nashville, TN	664 K	1.90 M	55.3%	27.2%	14.4%	1.9%	1449	693 K	157	50	25.0 K	19	4	99
Oklahoma City, OK	644 K	1.38 M	53.5%	14.1%	16.1%	0.5%	1124	650 K	49	21	8.3 K	19	9	65

a Data were from the 2015–2019 American Community Survey and all characteristics are for the city rather than metropolitan area.

b Data were from the American Public Transportation Association, Federal Transit Administration, and reports provided to the study by the transit agencies, and reflect 2019.

c Data were from the National Centers for Environmental Information and reflect 2019.

d Maximum temperature.zero-farezero-fare.

#### 2.2.2 Resident sample and procedures (Aim 2)

KCMO residents (Table 2) are being recruited from patients served by the University Health system, which is a large academic health system and safety-net provider in KCMO. The system comprises 10 hospital- and community-based primary care clinics located in the metropolitan area and serves a large portion of the city’s low-income residents. Patients are invited to participate if they reside in KCMO, are ≥18 years of age, speak English or Spanish, and had ≥1 visit to the health system between 3/1/2018 and 2/28/2020 and ≥ 1 visit between 4/1/2020 and 3/31/2022, which reflects the 2-year period before and after zero-fare began. Both visits must have included a weight and height measurement (to calculate BMI), not had a recorded pregnancy, and had a home address within the city limit. Recruitment spanned from December 2022 to December 2024, with a target goal of enrolling 480 bus users and ≥ 960 non-bus users. A total of 28,165 eligible patients were identified from the electronic health record and are being recruited through multiple contact methods (e.g., letters, phone calls, flyers). Participation involves completing a brief study survey to identify bus use. A subsample of 180 bus users matched to 180 non-bus users are invited to complete a second survey and an accelerometer assessment. Enrollment for this subsample is occurring from July 2023 to December 2024. Bus users are those who report using the KCATA city bus on ≥1 day during the past week. Propensity scores are used to identify matches (i.e., pairs of bus users and non-bus users to recruit into the subsample) based on sociodemographic characteristics and zip code as well as distance to the nearest bus stop, which are collected in the initial survey as detailed below. The propensity scores are computed using the R (34) package *MatchIt* (35).

**Table 2:** Characteristics of ZBT study participants.

	Mean (SD) or N (%)	
Characteristic	Health system sample^a^ (*N* = 2,435)^b^^,^^c^	
	Bus users (*N* = 381)	Non bus users (*N* = 819)
Age (y)		
18–29	17 (2.4)	67 (3.9)
30–44	133 (18.9)	328 (18.9)
45–59	287 (40.8)	598 (34.5)
60–74	243 (34.6)	619 (35.7)
75+	9 (1.3)	88 (5.1)
Missing	14 (2.0)	32 (1.8)
Sex at birth		
Female	453 (64.4)	1318 (76.1)
Male	248 (35.3)	407 (23.5)
No response Race/ethnicity	2 (0.3)	7 (0.4)
Black or African American	1098 (63.4)	491 (69.8)
White	457 (26.4)	129 (18.3)
Other	177 (10.2)	83 (11.8)
Bus use days/week	4.3 (2.2)	–

a All values reflect the baseline period prior to zero-fare (2018–2020).

b Reflects patients enrolled as of July 1, 2024.

c City bus use was not reported for 23 participants.

### 2.3 Measures

#### 2.3.1 Ridership and transit information (Aim 1)

The ridership data being collected from each city’s transit agency span March 1, 2017 to February 28, 2024, from 3 years prior to KCMO’s zero-fare policy to 4 years after. The data indicate, per each bus route, the number of unlinked passenger trips each month based on the number of riders boarding the bus. Yearly General Transit Feed Specification (36) data were then used to identify the route and associated bus stop locations to support geospatial analyses. At the end of the study period, a key informant from each transit agency is invited to complete a survey to capture more information about their transit system, including ridership tracking methods, bus fares, periods of zero-fare (e.g., start of pandemic), and major changes that occurred during the study period (e.g., new bus lines).

#### 2.3.2 Neighborhood environment variables

Neighborhood-level information on safety, built environment, and sociodemographic factors are being collected as detailed in Table 3. Variables were selected for inclusion based on their association with transit use or physical activity in prior research and availability and consistency across all included cities. All data except crime and crashes were collected from national sources. The crime and crash data were collected for each city with available data and processed to indicate the monthly number of crimes and crashes of different types. Geospatial analysis was performed to derive a version of each neighborhood variable that was specific to the included bus routes (Aim 1) and participant home addresses (Aim 2). The former set of variables were processed using a 500 m radial buffer around each bus stop in all included cities. The second set were based on a 500 m and 1 km street network buffer around each participant’s home.

**Table 3:** Neighborhood environment data in the ZBT study.

Topical area	Variables included
Macroscale features	
Socioedemographic^d^	Age; sex; education; race/ethnicity; racial/ethnic segregation; family households; female headed households with children.
Economic^d^	Median annual household income; poverty; households receiving public assistance; median home value; unemployment; income inequality; gentrification (reflecting 10-year changes)^e^.
Housing^d^	Rented housing; owner occupied housing; crowding; vacant housing; living in residence ≥1 year.
Walkability^f^	Residential density; retail/office/industrial/service/entertainment/health care/education/public administration density; land use mix; street connectivity; walkability index.
Transportation	Households with no vehicle^d^; take public transit to work^d^; proximity to transit stops^d^.
Crime^a^	Part I crimes against persons (assault, homicide, human trafficking, sex offenses); Part I crimes against property (arson, burglary, larceny/theft, motor vehicle theft, robbery); Part II crimes^b^ (vandalism, drug offenses, gambling, prostitution, weapons, loitering/vagrancy, disorderly conduct, driving under the influence, nonviolent family offences, liquor law violations, trespassing, kidnapping).
Crashes^c^	Number of vehicle crashes with a pedestrian or cyclist, categorized by severity: fatality; injury; neither.

a From city police reports 2017–2024, in accordance with the Federal Bureau of Investigation’s Uniform Crime Reporting Program (55) and following the National Incident-based Reporting System (56).

b KCMO only due to differences in crime reporting systems across cities.

c From state department of transportation reports 2017–2024.

d From American Community Survey (57) 2015–2019.

e Calculated based on American Community Survey data from 2005–2009 to 2015–2019.

f From U.S. Environmental Protection Agency (58) 2021.

#### 2.3.3 Participant surveys (Aim 2)

All participants are asked to complete Survey 1 (Table 4), which is primarily used to collect sociodemographic characteristics and bus use, and to identify participants to enroll into the 360-participant accelerometer subsample. Subsample participants are asked to complete Survey 2 to measure healthy eating (Aim 2) and factors to support exploratory analyses such as neighborhood perceptions, discrimination, destinations when using the bus, and perceptions of zero-fare.

**Table 4:** Self-reported health, neighborhood, and bus use factors measured in the ZBT study.

Survey and construct	Items	Description
Survey 1 (all participants)		
Sociodemographics	6	Age, sex at birth, gender identity, race, ethnicity, education, marital status,^a^ children in household,^a^ work status,^a^ and annual household income.^a^
Vehicle access^b^	1	Number of household drivable vehicles.
Transportation^b^	6	Days in past week used each mode of transportation: walking, biking, driving alone, passenger in vehicle, taxi/rideshare, and city bus.
Physical functioning^b^	1	How much physical health limits usual physical activities (1–5) (59).
Physical activity^a^	2	Days per week and minutes per day of moderate to strenuous physical activity on average over past month (60, 61).
Diet/nutrition^a^	1	Daily servings of fruits and vegetables on average over past month (0 to 6+) (62).
Employment or income changes^a^	1	Whether employment or income has changed since just before to the start of zero-fare (1 [much better] to 5 [much worse]).
Survey 2 (subsample)		
Diet/nutrition	4	Frequency in the past month consumed fruit, vegetables, snacks/sweets, (Never to 2 or more times per day) and sugar sweetened beverages (Never to 6 or more times per day) (63, 64).
Sources of food	6	Times per month different types of stores are visited to purchase food to prepare at home: supermarkets, superstores, corner stores, convenience stores, farmer’s markets, and food pantries (Never to 3+ times per month) (65).
Sources of prepared foods	5	Times per week different types of food outlets are visited to consume prepared food: pizza, fast food, fast-casual, full service restaurants, and convenience stores (Never to 3+ times per week) (65).
Meals prepared at home	1	Times in a typical week meals were prepared at home (0 to 10+).
Neighborhood disorder	8	Rating of seriousness of noise, traffic, food access, recreation areas, sidewalks, violence, trash, and off leash dogs (1–4) (66).
Neighborhood cohesion	5	Rating of agreement regarding neighbors being close-knit, willing to help each other, getting along, being trustworthy, and sharing values (1–5) (66).
Discrimination	7	Frequency being treated with less courtesy, receiving poorer service, being treated as not smart, people acting afraid, being treated unfairly by police, being threatened, and being followed in stores (never to almost every day) (67).
Bus use	9	Frequency of using the bus to get to/from work, friend’s/family’s houses, grocery stores, restaurants, health-related appointments, pharmacy, school, and other places (never to almost every day).
Perceptions of zero-fare	12	Rating of agreement on how policy has impacted participant in relation to access to different places, maintaining employment, riding more often, saving money, neighborhood safety, overcrowding of buses, and other impacts (1–5).

a Questions were framed to capture the current time period when the participant completed the survey.

b Questions were asked twice—the first was framed to capture the month prior to the start of the COVID-19 pandemic and the second was framed to capture the current time period when the participant completed the survey (2022 and after). The former questions referenced the COVID-19 pandemic because zero-fare started early into the pandemic and the start of the pandemic was a notable reference point for aiding recall.

#### 2.3.4 Device-measured physical activity and trips (Aim 2 subsample)

Participants in the accelerometer subsample are instructed to wear an ActiGraph wGT3X-BT accelerometer (ActiGraph LLC, Pensacola, FL) on the right iliac crest and a QStarz BT-Q1000XT GPS monitor (QStarz, Taipei, Taiwan) (37, 38) attached to the same belt for 7 days during waking hours. Accelerometer nonwear is determined using the Choi algorithm with a 90 min window, 30 min streamframe, and 2 min tolerance (39). Days with ≥10 h of wear time are considered valid days (40, 41). Moderate-to-vigorous physical activity (MVPA) is scored using the widely accepted Freedson 60s cut points for adults (42). The GPS records participants’ geo-coordinates (latitude and longitude) every 30s a GPS satellite signal is available. Trips are identified using the Physical Activity Location Measurement System (PALMS) trip classification algorithms, which have good validity for assessing pedestrian, bicycle, and vehicle trips (43, 44). To distinguish bus trips from other vehicle trips and identify pedestrian and cycling trips occurring just before or after each bus trip the GPS data will be integrated with transit logs, as has been done in prior research (3), and with geospatial data for bus routes and bus stop locations. Because transit logs may be incomplete for some participants, we aim to develop a decision tree approach for categorizing whether each trip is bus related (yes/no) using only GPS and bus stop location data. The approach will be evaluated using transit logs and visual inspections of a subset of data. The final derived variables include daily minutes spent in MVPA, bus trips, vehicle trips (excluding bus trips), walking trips, bus-related walking trips, cycling trips, and bus-related cycling trips.

#### 2.3.5 Transit log (Aim 2 subsample)

Participants in the accelerometer subsample also complete a transit log while wearing the study devices to record the number of bus trips they take each day, which will be integrated with the accelerometer and GPS data to identify missed and false trips.

#### 2.3.6 Health markers and economic barriers to health (Aim 2 all participants)

Upon the completion of enrollment, participant data will be obtained from all clinic visits occurring from March 1, 2017 to February 28, 2024. Height, weight, blood pressure, cardiometabolic biomarkers (i.e., insulin, blood glucose, hemoglobin A1c, high- and low-density lipoprotein cholesterol, and triglycerides), and economic barriers to health were collected by University Health providers during patient visits as part of usual care and obtained through the electronic health record. BMI was calculated as kg/m^2^. The economic barriers survey asked patients whether over the past 12 months they have (1) missed doctor’s appointments or going to the pharmacy because of transportation barriers, (2) skipped medications to save money, and (3) eaten less than they thought they should because there wasn’t enough money for food. All responses were recorded as yes/no.

### 2.4 Statistical considerations

#### 2.4.1 Analytic approach

Broadly, planned analyses involve two high-level considerations. First, inference of associations underlying each aim will be estimated utilizing generalized linear mixed models, to account for repeated timepoints (Aim 1), and nesting of participants within block groups (Aim 2). Second, synthetic controls will be generated specific to each aim for group-wise comparisons. This approach leverages a large sample of bus routes from non-zero-fare cities (Aim 1) and non-riders in KCMO (Aim 2) to create weighted (synthetic) control instances matched on baseline transit, neighborhood, and/or participant characteristics of the exposed/treatment group (45). The synthetic controls are used to account for differences between the cases and controls that arise from the quasi-experimental design. Additional models will be explored in which these baseline factors are adjusted as covariates.

To compare changes in bus ridership between Kansas City (zero-fare city) and non-zero-fare city bus routes in Aim 1, monthly route-level ridership will be modeled as a function of group (KCMO versus comparison cities), time (post- versus pre-zero-fare), and group × time interaction. Bus routes from the comparison cities, including their baseline ridership and macroscale neighborhood characteristics, will be used to create a synthetic comparison route for each KCMO bus route. Sensitivity analyses will be performed to comparing KCMO to each city individually and including baseline factors as covariates as opposed to creating synthetic controls.

To compare health indicators between zero-fare riders and non-riders in Aim 2, prospective analyses involve modeling participant BMI (primary), cardiometabolic markers, and economic barriers to health as a function of study group (zero-fare riders versus non-riders), time (post- versus pre-zero-fare), and group × time interaction. Among the accelerometer subsample, cross-sectional analyses (post-zero-fare) will model participant MVPA, travel behaviors, and healthy eating measures as a function of study group (zero-fare riders versus non-riders). Again, synthetic control and associated sensitivity analysis will be used based on the macroscale neighborhood factors shown in Table 3 and baseline participant characteristics collected in Survey 1.

#### 2.4.2 Power

The study was powered based on Aims 1 and 2. All analyses are powered at 80% with an alpha level of 0.05 for two-tailed tests. For Aim 1, with 53 bus routes in KCMO and 53 synthetic control routes, the minimal detectable effect is an increase in ridership in KCMO by 1,200 daily riders (3.6%) as compared to the comparison cities. This assumes a correlation of 0.5 between pre- and post-zero-fare ridership and an ICC = 0.25 reflecting the repeated time points within bus routes (84 months within each route).

For Aim 2, power analysis indicated 480 bus users and at least as many non-bus users need to be enrolled in the health system sample to detect a minimal effect size of d = 0.31 in changes between group means. Given a standard deviation of 2.6 in BMI changes over time based on prior transit research (15), this effect size reflects a minimal detectable difference BMI change between groups (bus users and non-bus users) of 0.81 units. This assumes 25% attrition by 4-years post-baseline, a correlation of 0.5 between pre and post BMI, and an ICC of 0.10 based on prior studies of BMI and physical activity in participants nested within block groups (46, 47).

The minimal detectable effect size for MVPA, which is only collected at a single time point post-baseline, is d = 0.43 for the health system subsample of 360 participants assuming an ICC of 0.10. Based on a standard deviation of 30 min/day from prior studies of transit and non-transit users (3, 16, 48), this effect size equates to a difference of 13 min/day of MVPA between bus users and non-bus users.

## 3 Discussion

This study builds upon previous health-focused natural experiment transit studies that have capitalized on a large-scale public transit interventions (14, 15, 18, 19, 25, 49). Each of these previous studies examined health impacts of an expanded transit line rather than a city-wide initiative and therefore only have the capacity to impact a subsample of the city population. To our knowledge the present study is the first in the US to examine the health impacts of a city-wide transit initiative through a natural experiment study. Because the current study is examining a zero-fare initiative, the positive impacts may be greater than previous studies as the intervention may be more likely to be utilized by low-income populations compared to fee for service initiatives. We are using similar accelerometer and GPS measures of physical activity to three of the aforementioned studies (15, 48, 49). Our study is enhanced by our approach using synthetic controls to more accurately reflect a comparable control group.

Similar zero-fare transit policy interventions have been conducted outside of the U.S., with Tallinn, Estonia as the largest city globally with citywide zero-fare transit. While there are differences in the two cities’ populations, we expect similar outcomes. These outcomes include increase ridership, increase mode share of public transit, and decrease miles traveled by car (25, 26) and an increase in demand for public transit (27). The policy was also linked with decreases in transportation inequity, showing increased mobility among lower income groups (28). The results of the present study will expand upon the available evidence for citywide zero-fare transit by presenting the impact on health markers.

Several other cities have implemented zero-fare interventions for sub-groups of the populations, including zero-fare for older adults and college/university students. In several studies, transit use increased (50–52). For older adults, past studies suggest that access to zero-fare transit increases physical activity (29, 52, 53) and improves overall wellbeing and social connection (29, 52). We expect similar results for these sub-populations from this project.

### 3.1 Strengths and limitations

Study strengths include the use of a large sample of controls (comparison cities and non-riders) to support rigorous synthetic control methods accounting for baseline differences between groups (45); the inclusion of neighborhood sociodemographic, environment, and transit factors; and the use of historical ridership and electronic health record data to enable pre-post evaluations. Limitations include the inability to collect individual-level device-based measures of baseline (before zero-fare was implemented) physical activity and healthy eating, lack of health data from individual participants in comparison cities, and overlap between the study period and COVID-19 pandemic, which impacted health behavior, transit, and economic factors (54).

## 4 Conclusion

Large-scale policy and environment systems approaches are needed to increase opportunities for incorporating active living and healthy eating into the daily routines of individuals from disadvantaged communities. The ZBT study will provide novel information on how zero-fare transit shapes population health through mechanisms related to improved economic factors, transportation, physical activity, and healthy eating among low-income groups. In doing so, the study aims to inform the prioritization of health impacts in public policy decision making.

## Data Availability

The raw data supporting the conclusions of this article will be made available by the authors, without undue reservation.
